# Effect of growth differentiation factor-15 (GDF-15) level on extent and severity of atherosclerosis in peripheral arterial disease patients free of obstructive coronary artery disease: A cross-sectional observational study

**DOI:** 10.1097/MD.0000000000044530

**Published:** 2025-09-19

**Authors:** Emrah Ozdemir, Murat Ziyrek

**Affiliations:** a Department of Cardiology, Biruni University Faculty of Medicine, Istanbul, Turkey; b Department of Cardiology, KTO Karatay University Faculty of Medicine, Konya, Turkey.

**Keywords:** atherosclerosis, GDF-15, TASC-2, ulcer

## Abstract

Members of the transforming growth factor-β superfamily, such as growth differentiation factor 15 (GDF-15), are potent regulators of vascular remodeling and play key roles in atherosclerosis. In this study, we investigated the effect of GDF-15 level on the extent and severity of atherosclerosis in patients with peripheral artery disease (PAD) without obstructive coronary artery disease. A total of 130 patients with PAD who were admitted to the cardiology outpatient department of Biruni University Health Center with complaints of chest pain and intermittent claudication, and whose coronary artery disease was excluded after coronary angiography, were included. GDF-15 levels were measured, and the TASC-2, Rutherford, and Fontaine classifications of patients with obstructive PAD patients were performed. Although there were no statistically significance between 5 Fontaine classes, 3 categories of Rutherford classification and 4 groups of TASC-2 in terms of BMI, platelet count, monocyte/HDL ratio, CRP, and GDF-15 levels were significantly increased. Furthermore, we also performed a regression analysis of parameters that reached statistical significance between the PAD(+) and PAD(−) groups to analyze the independent risk factors for obstructive PAD and lower extremity ulcers. Although none of the mentioned parameters were found to be independent risk factors for obstructive PAD, CRP ([*P* = .006, β: 1.098, OR [(95% CI): 1.027–1.174]) and GDF-15 ([*P* = .002, β: 1.002, OR [(95% CI): 1.004–1.010]) were shown to be independent risk factors for lower- extremity ulcers. Our prospective study postulated that CRP and GDF-15 levels are elevated in patients with obstructive PAD and they are independent risk factors for lower-extremity ulcers.

## 1. Introduction

Atherosclerosis, which is chronic inflammatory state of the vascular intima, usually affects the branching points of the vascular tree where shear stress changes occur.^[1]^ It is characterized by impaired equilibrium between the inflammatory response and antioxidant mechanisms.^[2]^ It is one of the leading causes of occlusive arterial diseases, including ischemic heart disease, ischemic stroke, and peripheral arterial disease (PAD).^[3]^

Growth differentiation factor 15 (GDF-15) is a major member of the transforming growth factor-β superfamily with little structural similarity to other members of the group.^[4]^ Transforming growth factor-β family members are potent regulators of vascular remodeling and play key roles in atherosclerosis.^[5]^ Various cell types including macrophages, adipocytes, and endothelial and vascular smooth muscle cells, which play key roles in inflammatory processes and atherosclerosis, express GDF-15.^[6]^ GDF-15 is a stress-inducible cytokine that can be upregulated by various inflammatory or stress-induced proteins, such as tumor necrosis factor-α, interleukin-2, and macrophage colony stimulating factor-1.^[4]^ Hence, various cardiometabolic conditions, including atherosclerosis, endothelial dysfunction, heart failure, insulin resistance, diabetes mellitus, and obesity, are associated with increased GDF-15 levels.^[4]^ Furthermore, circulating GDF-15 levels can be used to predict cardiovascular disease progression^[7]^ and, cardiac/noncardiac, and all-cause mortality.^[8,9]^

Peripheral arterial disease (PAD) is now considered a global pandemic, with more than 200 million affected individuals in different locations due to increased age and exposure to cardiovascular risk factors.^[10]^ Various studies have shown that cardiovascular mortality and morbidity are increased in patients with symptomatic or asymptomatic PAD, patients even after adjustment for conventional risk factors.^[11]^

In our study, we analyzed the effect of GDF-15 levels on the extent and severity of PAD in patients without coronary artery disease (CAD).

## 2. Material and methods

### 2.1. Study design and population

This was a single-center prospective cross-sectional study. A total of 411 patients with stable angina pectoris and intermittent claudication who underwent coronary and peripheral angiography between March 1, 2021 and August 31, 2022, were investigated. Patients with obstructive CAD (atherosclerotic coronary artery lesions causing stenosis ≥50% in any vessel on coronary angiography, accepted as obstructive CAD), previous history of coronary and/or peripheral arterial percutaneous interventions, coronary and/or peripheral arterial bypass grafting operations, active infection, chronic kidney disease (stage 4 or higher), active cancer, or severe hepatic dysfunction were excluded. A total of 130 patients without obstructive CAD were included in this study.

### 2.2. Study protocol

Detailed medical histories, clinical-demographic characteristics, and the presence of cardiovascular risk factors were recorded. A detailed physical examination of the lower-extremity ulcers was performed. Subsequently, venous blood samples were collected, and peripheral angiographic evaluations of all patients were performed. Two groups were formed from the included patients based according to the presence or absence of obstructive PAD. Written informed consent was obtained from all the patients. All relevant data are presented in with the paper and its supporting information files.

### 2.3. Ethical statement

This study was conducted in accordance with the principles of the Declaration of Helsinki. All methods were performed according to the relevant guidelines. Our study was approved by the Biruni University Faculty of Medicine Ethics Committee (approval no. 2020/46-02).

### 2.4. Biochemical analysis

Venous blood samples were collected from all patients were taken after 8 to 12 hours of fasting. Serum and plasma samples were obtained by centrifugation at 3000 ×g for 15 minutes at 4°C. Immediately after centrifugation, the plasma samples were stored at −80°C for GDF-15 analysis. Fasting blood glucose, total cholesterol, high-density lipoprotein (HDL), triglyceride (TG), low-density lipoprotein, blood urea nitrogen, creatinine, complete blood count, aspartate amino transferase, and alanine amino transferase levels were analyzed using a Roche Hitachi Cobas 6000 system (Roche Diagnostics, GmbH, Mannheim, Germany) andusing enzymatic methods. Plasma GDF-15 levels were analyzed using a commercially available ELISA kit (Human GDF-15 Quantikine ImmunAssay, number DGD150; R&D Systems, Minneapolis, MN).

### 2.5. Angiographic evaluation

All angiographies (coronary and peripheral) were performed and interpreted by interventional cardiologists with more than ten years of working experience. Two experienced cardiologists analyzed all angiographic data. Normal peripheral arteries or atherosclerotic peripheral artery lesions causing stenosis of <50% in any vessel on peripheral angiography were considered nonobstructive PAD. The TransAtlantic Inter-Society Consensus for the Management of Peripheral Arterial Disease-II (TASC-II),^[12]^ Rutherford,^[13]^ and Fontaine^[14]^ classifications of obstructive PAD were used performed.

## 3. Statistical analysis

In this study, continuous variables are expressed as mean ± SD, and categorical variables are expressed as counts and percentages. Fitness to normal distribution was analyzed using the Kolmogorov–Smirnov test. Homogeneity of variance was calculated using the Levene test and the Lilliefors significance correction test. Continuous variables were compared using either Student *t* test or Mann–Whitney *U* test, and continuous variables were compared using either Chi-square or Fisher’s exact tests. Pearson’ sor’s its nonparametric counterpart, Spearman’s correlation analysis, was performed to evaluate the correlations between continuous variables. Linear logistic regression analysis was performed to explore independent factors associated with obstructive PAD skin ulcers. One-way analysis of variance (ANOVA) or its nonparametric counterpart Kruskal–Wallis tests were used to analyze intergroup differences. Tukey’s HSD or Games-Howell tests were used for post hoc analysis of parametric data, and the Mann–Whitney *U* test was used for post hoc analysis of nonparametric data. Binary regression analysis was performed to explore independent factors associated with obstructive PAD and skin ulcers. Receiver operating characteristic curve analysis was used to test the diagnostic accuracy of GDF-15 and CRP levels for the prediction of obstructive PAD and skin ulcers. Statistical significance was set at *P* < .05. All analyses were performedconducted using a commercially available software package (SPSS version 16.0; SPSS, Chicago, IL).

## 4. Results

A total of 130 (68 male, 62 female) patients with a mean age of 57.11 ± 12.07 were included. Of these 130 patients, 79 had obstructive PAD and formed the PAD(+) group, and 51 did not have obstructive PAD and formed the PAD(−) group. When we analyzed the demographic and clinical data of both groups, the BMI was significantly lower (28.34 ± 2.56 vs 31.23 ± 6.93; *P* < .001), hyperlipidemia (62.02% vs 29.41%; *P* < .001), smoking (83.54% vs 5.88%; *P* < .001), and lower extremity ulcers (29.11% vs 0%; *P* < .001) were significantly higher in the PAD(+) group. Furthermore, biochemical analysis revealed that; HDL level (40.82 ± 9.11 vs 48.53 ± 9.69; *P* < .001) and PLT count (252.48 ± 87.55 vs 285.89 ± 71.12; *P* = .032) were significantly lower, CRP (8.11 ± 1.56 vs 4.25 ± 1.34; *P* < .001), monocyte/HDL ratio (0.215 ± 0.079 vs 0.184 ± 0.052; *P* = .016), and GDF-15 level (2403.38 ± 428.38 vs 655.27 ± 131.62; *P* < .001) were significantly higher in PAD(+) group. Detailed demographic, clinical, and biochemical data for both the groups are shown in Table 1.

**Table 1:** Baseline demographical, clinical, and biochemical characteristics of PAD(+) and PAD(−) groups are given.

Variables	PAD(+) group (n = 79)	PAD(−) group (n = 51)	*P*
Age (yr)	57.41 ± 12.61	56.65 ± 11.24	.727
Gender (male n/%)	44/55.69	26/50.98	.372
BMI (kg/m^2^)	28.34 ± 2.56	31.23 ± 6.93	**<.001**
Hypertension (n/%)	48/60.75	36/70.58	.170
Hyperlipidemia (n/%)	49/62.02	15/29.41	**<.001**
Diabetes mellitus (n/%)	32/40.50	21/41.17	.542
Smoking (n/%)	66/83.54	3/5.88	**<.001**
Family history (n/%)	8/10.12	6/11.76	.160
Lower extremity ulcer (n/%)	23/29.11	0	**<.001**
Fasting blood glucose (mg/dL)	102 (79–330)	102 (75–286)	.934
Total cholesterol (mg/dL)	185.86 ± 51.16	195.00 ± 25.16	.243
LDL (mg/dL)	111.46 ± 36.49	117.02 ± 25.47	.344
HDL (mg/dL)	40.82 ± 9.11	48.53 ± 9.69	**<.001**
Triglyceride (mg/dL)	164 (47–1420)	167 (53–755)	.486
BUN (mg/dL)	29 (16-93)	30 (14–57)	.862
Creatinine (mg/dL)	0.79 (0.68–1.72)	0.78 (0.65–1.67)	.114
Sodium (mEq/L)	137.99 ± 3.47	138.88 ± 2.38	.114
Potasium (mEq/L)	4.34 ± 0.43	4.25 ± 0.32	.179
AST (mg/dL)	22 (9–46)	24 (9–48)	.192
ALT (mg/dL)	23 (7–83)	24 (7–69)	.372
CRP (ng/mL)	8.11 ± 1.56	4.25 ± 1.34	**<.001**
Hemoglobin (g/dL)	13.18 ± 1.95	13.54 ± 1.44	.267
WBC (10^9^/L)	8.89 ± 2.23	8.34 ± 2.64	.126
PLT (10^9^/L)	252.48 ± 87.55	285.89 ± 71.12	**.032**
Neutrophil (%)	63.35 (35–82)	60 (37–85)	.468
Monocyte (%)	8.26 ± 2.19	8.55 ± 1.86	.433
Monocyte/HDL ratio	0.215 ± 0.079	0.184 ± 0.052	**.016**
GDF-15 (pg/mL)	2403.38 ± 428.38	655.27 ± 131.62	**<.001**

ALT = alanine aminotransferase, AST = aspartate aminotransferase, BMI = body mass index, BUN = blood urea nitrogen, CRP = C-reactive protein, HDL = high-density lipoprotein, LDL = low-density lipoprotein, PLT = platelet, WBC = white blood cell. The bold values indicate statistically significant *P < .05.

### 4.1. Evaluation of extent and severity of atherosclerosis in PAD

**Figure 1.**
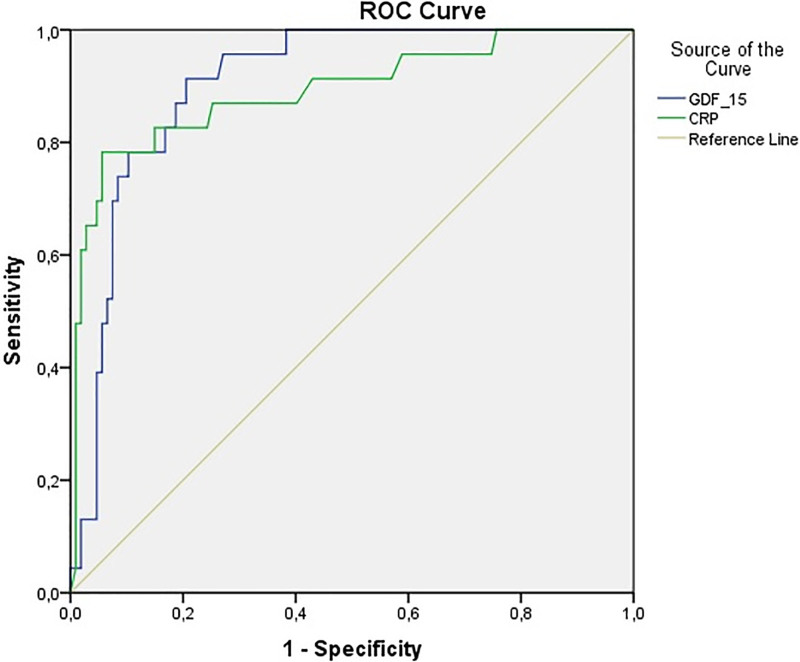
ROC (Receiver Operating Characteristic) curve analysis of the performance of the CRP and GDF-15 levels for diagnosing lower extremity ulcers. CRP = C-reactive protein, GDF-15 = growth differentiation factor-15.

PAD(+) was further classified according to the Fontaine, Rutherford, and TASC-II PAD classification systems. The parameters that reached statistical significance between the PAD(+) and PAD(−) groups were reevaluated. Although there was no statistically significant difference in terms of HDL, platelet count, monocyte/HDL ratio and BMI in Fontaine, Rutherford and TASC-II classifications, CRP and GDF-15 levels were found to increase significantly in proportion to the severity of PAD. Detailed inter-group analysis data are presented in Table 2. Furthermore, we also performed a regression analysis of parameters that reached statistical significance between the PAD(+) and PAD(−) groups to analyze the independent risk factors for obstructive PAD and lower extremity ulcers. Although none of the mentioned parameters were found to be independent risk factors for obstructive PAD, CRP ([*P* = .006, β: 1.098, OR [(95% CI): 1.027–1.174] ) and GDF-15 ([*P* = .002, β: 1.002, OR [(95% CI): 1.004–1.010] )were shown to be independent risk factors for lower- extremity ulcers. Subsequently, we performed receiver operating characteristic analysis to determine the cutoff values for CRP and GDF-15 levels to detect lower-extremity ulcers. The area under the curve for CRP was 0.892 [%95 CI: 0.807–0.977] and GDF-15 was 0.905 [%95 CI: 0.851–0.959]. A cutoff value of 8.65 ng/mL for CRP level was associated with 82.6% sensitivity and 82.2% specificity, and a cutoff value of 2331 pg/mL for GDF-15 level was associated with 82.8% sensitivity and 82.4% specificity in predicting lower-extremity ulcers (Fig. 1).

**Table 2:** Comparison of various parameters according to different PAD classification systems.

Parameter	Class-1 (n = 17)	Class-2a (n = 15)	Class-2b (n = 16)	Class-3 (n = 15)	Class-4 (n = 16)	*P*
Fonteine classification						
BMI	28.76 ± 3.45	28.97 ± 4.13	27.75 ± 1.41	28.95 ± 3.42	28.63 ± 2.70	.288
HDL (mg/dL)	43.23 ± 3.78	41.95 ± 4.39	43.67 ± 5.43	44.53 ± 3.91	42.05 ± 7.61	.117
PLT (10^9^/L)	254.75 ± 45.11	253.65 ± 32.76	251.76 ± 43.28	252.56 ± 42.83	256.62 ± 56	.236
Monocyte/HDL ratio	0.218 ± 0.069	0.229 ± 0.093	0.215 ± 0.081	0.221 ± 0.078	0.211 ± 0.079	.581
CRP (ng/mL)	8.24 ± 2.27	9.16 ± 3.42	10.52 ± 2.81	16.85 ± 4.46	28.64 ± 5.29	**<.001** *
GDF-15 (pg/mL)	1534.14 ± 412.23	1856.42 ± 511.21	2212.90 ± 596.12	2579.33 ± 571.67	3030.35 ± 623.45	**<.001** †

Parameter		Category-I (n = 34)	Category-II (n = 24)		Category-III (n = 21)	*P*
Rutherford classification						
BMI		29.84 ± 5.67	28.92 ± 3.36		28.59 ± 2.58	.494
HDL (mg/dL)		44.60 ± 5.41	43.81 ± 3.69		42.85 ± 4.13	.434
PLT (10^9^/L)		264.63 ± 38.91	260.19 ± 43.09		262.54 ± 45.82	.744
Monocyte/HDL ratio		0.202 ± 0.070	0.198 ± 0.047		0.203 ± 0.071	.458
CRP (ng/mL)		5.45 ± 2.18	12.64 ± 4.25		29.11 ± 6.52	**<.001** ‡
GDF-15 (pg/mL)		1258.81 ± 352.17	2273.69 ± 465.41		3067.25 ± 507.17	**<.001** §

Parameter	Type-A (n = 17)	Type-B (n = 15)	Type-C (n = 20)		Type-D (n = 27)	*P*
TASC-2 classification						
BMI	29.40 ± 2.82	28.83 ± 3.83	27.99 ± 2.07		28.24 ± 2.47	.588
HDL (mg/dL)	37.57 ± 7.15	40.50 ± 6.98	39.27 ± 6.47		42.33 ± 7.810	.634
PLT (10^9^/L)	246.14 ± 53.59	250.43 ± 61.93	246.73 ± 52.76		251.49 ± 45.42	.969
Monocyte/HDL ratio	0.235 ± 0.033	0.239 ± 0.029	0.231 ± 0.053		0.236 ± 0.057	.187
CRP (ng/mL)	8.46 ± 2.34	8.18 ± 3.12	8.94 ± 2.95		20.90 ± 5.24	**.011** ∥
GDF-15 (pg/mL)	1325.00 ± 187.44	1771.90 ± 307.96	2104.45 ± 410.61		2914.38 ± 528.12	**<.001** ¶

BMI = body mass index, CRP = C-reactive protein, HDL = high-density lipoprotein, PLT = platelet. The bold values indicate statistically significant *P < .05.

* Class-1 versus class-3; *P* < .001 – class-1 versus class-4; *P* < .001 – class-2a versus class-3; *P* = .007 – class-2a versus class-4; *P* < .001 – class-2b versus class-3; *P* = .045 – class-2b versus class-4; *P* < .001.

† Class-1 versus class-2a; *P* = .003 – class-1 versus class-2b; *P* < .001 – class-1 versus class-3; *P* < .001 – class-1 versus class-4; *P* < .001 – class-2a versus class-3; *P* < .001 – class-2a versus class-4; *P* < .001 – class-2b versus class-4; *P* < .001 – class-3 versus class-4; *P* = .031.

‡ Category-I versus category-II; *P* = .014 – category-I versus category-III; *P* < .001 – category-II versus category-III; *P* < .001.

§ Category-I versus category-II; *P* < .001 – category-I versus category-III; *P* < .001 – category-II versus category-III; *P* = .003.

∥ Type-A versus type-D; *P* = .005 – type-C versus type-D; *P* = .012.

¶ Type-A versus type-B; *P* = .041 – type-A versus type-C; *P* = .005 – type-A versus type-D; *P* < .001 – type-B versus type-D; *P* < .001 – type-C versus type-D; *P* < .001.

## 5. Discussion

The major finding of this study was that the GDF-15 and CRP levels were significantly higher in patients with obstructive PAD. Furthermore, although GDF-15 and CRP levels are not independent predictors of obstructive PAD, they have been shown to be independent risk factors for lower extremity ulcers. In addition, we determined the cutoff values for GDF-15 and CRP with higher sensitivity and specificity for the prediction of lower-extremity ulcers.

Peripheral arterial disease is primarily caused by the buildup of fatty atherosclerotic plaques in the lower extremities. Both CAD and PAD result from the same pathophysiological cascades at different locations of the arterial tree locations. Well-known atherosclerotic risk factors such as hyperlipidemia, diabetes mellitus, hypertension, and smoking are also associated with PAD.^[15]^ In addition, an association between PAD and an elevated risk of cardiovascular morbidity and mortality has been previously reportedshown. In a recent study, Heffron et al reported that although increased BMI is an independent risk factor for PAD in women, underweight and men with a normal BMI exhibited a higher prevalence of PAD than overweight and obese men.^[16]^ According to the aforementioned study, the reason for the sex differences in PAD prevalence is not clear, and maternal placental syndromes could be one of the suggested mechanisms. Similarly, in our study, we also found that BMI was significantly lower in the PAD(+) group, in which the male sex was dominant. Furthermore, although hyperlipidemia and smoking were significantly higher in the PAD(+) group, as expected, there was no difference in the prevalence of diabetes mellitus between the groups in our study. It has been previously shown that, among various lipid measures, low HDL and high triglyceride levels are associated with incident PAD.^[17]^ We also found that the HDL levels were significantly lower in the PAD(+) group. However, we did not find any significant differences in triglyceride levels. Although previous studies have shown that increased mean platelet volume is an important marker of platelet activity^[18]^; therefore, larger platelets are more active and more thrombotic than smaller ones,^[19]^ there isare hardly any data analyzing the relationship between platelet count and PAD. In our study, we showed that platelet count was significantly lower in patients with obstructive PAD. This could be the result of the antiplatelet drugs used by patients with PAD(+).

Inflammatory markers are elevated in atherosclerotic processes, including PAD, andAs inflammation is implicated in the development and progression of atherosclerosis, inflammatory markers are elevated in atherosclerotic processes, including PAD. Hence, Shankar et al showed that higher CRP levels were significantly associated with PAD in patients without cardiovascular diseases.^[20]^ Furthermore, and in a different study, Vu et al reported that the likelihood of PAD is enhanced by elevated CRP levels in patients with metabolic syndrome and DM.^[21]^ In both studies, PAD was diagnosed noninvasively using ankle brachial index. In our study, we showed that the CRP levels were significantly higher in patients with invasively diagnosed PAD. Although the CRP level was not found to be an independent risk factor for obstructive PAD, CRP was an independent risk factor for lower-extremity ulcers, and we also provided a relevant cutoff value of CRP for the diagnosis of lower-extremity ulcers. GDF-15, a member of the TGF family, is also involved in inflammatory processes and apoptosis. Higher levels of GDF-15 induced by hypoxia promote angiogenesis,^[22]^ and GDF-15 maymight be an important factor in local angiogenesis. Consistently, it was previously shown in another study showed that high GDF-15 levels were associated with an increased risk of amputation and/or death in patients with PAD.^[23]^ In our study, we also found that GDF-15 levels were significantly higher in obstructive PAD. However, it was found to be the only independent risk factor for lower-extremity ulcers and provided a reliable cutoff value for the diagnosis of lower-extremity ulcers. Our data support the previous literature.

### 5.1. Limitations of study

The limitations of our study include its single-center design and relatively small sample size. Larger multicenter studies are warranted to better determine the role of GDF-15 in the diagnosis, treatment, and assessment of PAD severity of PAD, larger multicenter studies are warranted.

## 6. Conclusion

In this prospective study, we showed that CRP and GDF-15 levels were elevated in patients with obstructive PAD patients and were independent risk factors for lower-extremity ulcers. Furthermore, this study provides reliable cutoff values forof CRP and GDF-15 for the diagnosis of lower-extremity ulcers.

## Author contributions

**Conceptualization:** Emrah Ozdemir, Murat Ziyrek.

**Data curation:** Emrah Ozdemir.

**Formal analysis:** Emrah Ozdemir.

**Investigation:** Emrah Ozdemir.

**Methodology:** Emrah Ozdemir.

**Project administration:** Emrah Ozdemir, Murat Ziyrek.

**Resources:** Emrah Ozdemir.

**Software:** Emrah Ozdemir.

**Supervision:** Emrah Ozdemir.

**Validation:** Emrah Ozdemir, Murat Ziyrek.

**Visualization:** Emrah Ozdemir, Murat Ziyrek.

**Writing – original draft:** Emrah Ozdemir.

**Writing – review & editing:** Emrah Ozdemir, Murat Ziyrek.
